# Rutin ameliorates carbon tetrachloride (CCl_4_)-induced hepatorenal toxicity and hypogonadism in male rats

**DOI:** 10.7717/peerj.7011

**Published:** 2019-05-29

**Authors:** Hany Elsawy, Gehan M. Badr, Azza Sedky, Basem M. Abdallah, Abdullah M. Alzahrani, Ashraf M. Abdel-Moneim

**Affiliations:** 1 Department of Chemistry, Faculty of Science, King Faisal University, Al-Hofuf, Al-Ahsa, Saudi Arabia; 2 Department of Chemistry, Faculty of Science, Tanta University, Tanta, Egypt; 3 Department of Biological Sciences, Faculty of Science, King Faisal University, Al-Hofuf, Al-Ahsa, Saudi Arabia; 4 Department of Zoology, Faculty of Science, Ain Shams University, Cairo, Egypt; 5 Department of Zoology, Faculty of Science, Alexandria University, Alexandria, Egypt; 6 Endocrine Research (KMEB), Department of Endocrinology, Odense University Hospital and University of Southern Denmark, Odense, Denmark

**Keywords:** Carbon tetrachloride, Hepatorenal failure, Rutin, Hypogonadism, Dyslipidemia, Oxidative stress

## Abstract

Rutin, a food derived-polyphenolic bioflavonoid, has been acknowledged for several health benefits. This study aims to explore the ameliorative effects of rutin against carbon tetrachloride (CCl_4_) toxicity in male rats. Adult male rats were given either CCl_4_ (30% in olive oil, 3 ml/kg b.w. intraperitoneally) alone or in combination with rutin (70 mg/kg intragastrically) twice a week for 4 weeks. Our data showed that rutin mitigated CCl_4_ hepatorenal damage, as indicated by diagnostic markers (i.e., transaminases, alkaline phosphatase, total bilirubin, total protein, albumin, urea, uric acid and creatinine), and histopathological findings. In addition, CCl_4_ induced profound elevation of free radical generation and oxidative stress, as evidenced by increasing lipid peroxidation and reducing catalase, superoxide dismutase and glutathione peroxidase activities in liver, kidney and testicular tissues; these effects were suppressed by coexposure with rutin. Moreover, the increase in the levels of serum triglycerides, cholesterol, low-density lipoprotein cholesterol, and very-low-density lipoprotein cholesterol induced by CCl_4_ was effectively counteracted by rutin. The decrease in the level of high-density lipoprotein cholesterol in the CCl_4_ group was also counteracted by rutin treatment. Interestingly, the decreased levels of hormonal mediators associated with sperm production, including serum testosterone, luteinizing hormone and follicle-stimulating hormone, and the impaired sperm quality induced by CCl_4_ were reversed by rutin. Data from the current study clearly demonstrated that rutin supplementation could at least partly overcome CCl_4_-induced hepatotoxicity, nephrotoxicity and reproductive toxicity by antioxidant and antidyslipidemic effects.

## Introduction

Industrial progress has a poisonous nature. CCl_4_ is one of the most potent environmental contaminants (Hefnawy & Ramadan, 2013). Humans are exposed to CCl_4_ via oral, inhalation and dermal routes (Manibusan, Odin & Eastmond, 2007). CCl_4_ intoxication is associated with high free radical production in several organs, including the liver and kidney (Ozturk et al., 2003; Preethi & Kuttan, 2009). CCl_4_ binds to liver cytochrome P450 to form trichloromethyl (CCl_3_) free radicals, which initiate membrane lipid peroxidation (Abdel-Kader et al., 2018). Secondary metabolic radicals of CCl_4_, such as trichloromethylperoxy radical (CCl_3_O_2_), react with lipids or proteins. This alters the permeability of the mitochondria, endoplasmic reticulum, and plasma membrane, resulting in cell damage (Rahman et al., 2017). CCl_4_-induced damage also includes altering the endogenous antioxidants in tissues Alshammari, Balakrishnan & Chinnasamy (2017), which is manifested by histopathological lesions. Meanwhile, some researchers reported that the administration of CCl_4_ elevated cholesterol, triglycerides, and free fatty acids in the liver and kidney of rats (Marimuthu et al., 2013) and caused male genotoxic effects in mouse bone marrow and germ cells (Diab et al., 2018).

Antioxidants are vital substances that possess the ability to protect the body from damage caused by free radical-induced oxidative stress (Abdel-Moneim et al., 2015). Much attention is paid to the protective effects of natural antioxidants against chemically induced toxicities (Frei & Higdon, 2003). Among these compounds, flavonoids are plant-based polyphenols that exhibit a wide range of beneficial effects in a multitude of disease models (see Mahmoud et al., 2019 for review). Rutin (named vitamin P) is composed of flavonol quercetin and disaccharide rutinose (Ganeshpurkar & Saluja, 2017). It is an important constituent of food and beverages. Many studies have suggested that rutin exerts antioxidant, anti-inflammatory, anti-apoptotic, antihyperuricemic, and antihyperlipidemic properties (Hosseinzadeh & Nassiri-Asl, 2014; Gelen et al., 2017; Radwan & Abdel Fattah, 2017; Khajevand-Khazaei et al., 2018). It has been reported that rutin prevented methotrexate-induced hepatic injury in a rat model (Erdogan et al., 2015). Moreover, the use of rutin for the treatment of hyperglycemic complications has been documented (Ghorbani, 2017). Rutin also showed a promising effect against oxidative insult associated with male infertility (Mehfooz et al., 2018). Therefore, the present work investigates the antioxidant and anti-hyperlipidemic effects of oral rutin administration as potential mechanisms driving its therapeutic ability against CCl_4_-induced hepatorenal and testicular toxicity.

## Material and Methods

### Chemicals and reagents

Carbon tetrachloride CCl_4_ was purchased from Sigma Chemicals (St. Louis, MO, USA). Rutin was purchased from Loba Chemie (Mumbai, India). All other reagents were of analytical grade.

### Animals and experimental design

Male albino rats weighing 180–200 g were used in the present study. All experimental procedures were reviewed and approved by the research ethics committee at King Faisal University (Ref. No. KFU-REC/2019-03-01). Rats were maintained under standard laboratory conditions and had fed chow and water *ad libitum*. After 2 weeks of acclimatization, the animals were randomly divided into four groups (five rats in each). Group 1 served as the control and received only vehicles, i.e., 1 ml/kg b.w. saline intragastrically and olive oil (3 ml/kg b.w.) intraperitoneally twice a week for 4 weeks. Group 2 (CCl_4_) was administrated CCl_4_ (30% in olive oil) at a dose of 3 ml/kg b.w. intraperitoneally twice a week for 4 weeks. Group 3 (rutin) received rutin in normal saline at a dose of 70 mg/kg b.w. intragastrically twice a week for 4 weeks. Group 4 (rutin + CCl_4_) received rutin at a dose of 70 mg/kg b.w. intragastrically, in addition to CCl_4_ administration, twice a week for 4 weeks. Doses of CCl_4_ and rutin were selected as previously reported by Khan, Khan & Sahreen (2012). After 24 h of the last treatment, animals were sacrificed, and blood samples were drawn from the dorsal aorta into dry glass centrifuge tubes, left to clot and then centrifuged at 5,000 rpm for 10 min. Serum was separated and stored at −80 °C for further assays. Small parts of the liver, kidney and right testes were washed in ice-cold normal saline solution and frozen at −80 °C for future analysis. For histological studies, other portions of the liver and kidney were quickly removed and fixed in an appropriate fixative.

### Assay of liver markers

Alkaline phosphatase (ALP), aspartate aminotransferase (AST) and alanine aminotransferase (ALT) enzymes and total protein were estimated by commercial kits (ALP; REF: ALP101050, AST; REF: GOT111120, ALT; REF: GPT1131001, total protein; REF: TP116150; BIOMED Diagnostics, Oberschleißheim, Germany). AST and ALT were measured by the decrease in absorbance at 340 nm, which is directly proportional to the oxidation of NADH to NAD. ALP activity was determined by measuring the per time absorption increase at 405 nm. Total bilirubin was measured using a Diamond Diagnostics kit, Germany (REF: BIL099100). Albumin was determined by the modified bromocresol green colorimetric method using its relevant kit (REF: 210 002, SPECTRUM, Egypt).

### Assay of kidney markers

Urea and creatinine acid were estimated using commercial kits (urea; REF: URE118200, creatinine; REF: CRE106240; BIOMED Diagnostics, Oberschleißheim, Germany). Briefly, serum urea was enzymatically determined and colorimetrically measured, where the conversion of urea in the sample by urease enzyme provided a colored complex that can be measured by spectrophotometry at 578 nm. Creatinine was determined by standard alkaline picric acid method, where creatinine reacts with picric acid in an alkaline medium to give a yellow-orange color complex, which was measured at 492 nm. The intensity of the color is proportional to the concentration of the creatinine concentration. The uric acid kit was obtained from SPECTRUM, Egypt (REF: 323001). Uric acid level was estimated colorimetrically by the dual action of uricase and peroxidase enzymes in the presence of 4-amino-antipyrine. The formed red-color quinoneimine dye was recorded at 546 nm.

### Hepatic and renal histopathology

Liver and kidney tissues were fixed in 10% neutral formalin solution for 24 h, dehydrated in ascending series of ethanol, cleared in xylene then embedded in paraffin wax. Sections were stained with conventional haematoxylin and eosin (H&E) dye and examined using a Nikon 80i light microscope (Nikon Corporation, Tokyo, Japan).

### Estimation of tissue malondialdehyde (MDA) and antioxidant enzymes

For homogenate preparation, the desired tissues were quickly removed, cleaned and washed in ice-cold saline. They were finely minced and homogenized in 0.1 M phosphate buffer pH 7.4 using glass Teflon homogenizer. The homogenate was centrifuged at 6,000 rpm for 30 min at 40 °C then the supernatant was used for biochemical estimations.

MDA levels and superoxide dismutase (SOD), catalase (CAT) and glutathione peroxidase (GPx) enzyme activities were assayed according to the manufacturer’s protocol (MDA; CAT No.: MD 2528, SOD; CAT. No.: SA 2520, CAT; CAT. No.: CA 2516; GPx; CAT. No.: GP 2524; Biodiagnostic, Dokki, Egypt). Principally, the colorimetric assay of MDA involves the reaction of MDA with thiobarbituric acid (TBA) in acidic medium at 95 °C to form thiobarbituric acid reactive product, and the absorbance of the resultant pink product can be measured at 534 nm. SOD assay relies on the ability of the SOD to inhibit the phenazine methosulphate-mediated reduction of nitro-blue tetrazolium dye. CAT assay colorimetric method depends on the measurement of the hydrogen peroxide (H_2_O_2_) substrate remaining after the action of CAT present in the sample. The rest of H_2_O_2_ reacts with 3,5-dichloro-2-hydroxybenzene sulfonic acid and 4-aminophenazone to yield a colored chromophore with a color intensity that is inversely proportional to the amount of CAT in the sample. The activity of GPx was measured indirectly, based on the principle that oxidized glutathione (GSSG), produced upon reduction of an organic peroxide by GPx, is immediately recycled to its reduced form (GSH) by glutathione reductase (GR). This is accompanied by oxidation of NADPH (GR coenzyme) to NADP+, which was monitored by the decrease in absorbance at 340 nm. The protein contents of the tissue samples (i.e., liver, kidney and testis) were determined by the method of Lowry et al. (1951) using bovine serum albumin as a standard.

### Estimation of serum lipid profile

Total cholesterol (TC), triglyceride (TG), and high-density lipoprotein cholesterol (HDL-C) levels were determined by using commercial kits (TC; CAT. No.: CH 1220, TG; CAT. No.: TR 2030, HDL-C; CAT. No.: CH 1230; Biodiagnostic, Dikka, Egypt). Very-low-density lipoprotein cholesterol (VLDL-C) and low-density lipoprotein cholesterol (LDL-C) were calculated using Friedewald’s formula (Friedewald, Levy & Fredrickson, 1972).

### Estimation of reproductive hormones

Serum testosterone was measured using a rat testosterone ELISA kit (CAT. No. KT-29533) purchased from K-assay, WA, USA. The levels of luteinizing hormone (LH) were determined by using Shibayagi’s rat LH ELISA kit, while the levels of follicle stimulating hormone (FSH) were estimated by using a rat FSH ELISA kit obtained from Biovendor, Tokyo, Japan. All hormonal assays employ the competitive inhibition enzyme immunoassay technique. Briefly, a monoclonal antibody specific for the estimated hormone has been pre-coated onto a microplate. A competitive inhibition reaction was started between biotin labeled hormone and the unlabeled one in our samples with the pre-coated antibody specific for the hormone. After incubation, the unbound conjugate was washed off. Then, avidin conjugated to horseradish peroxidase (HRP) was added to each well and incubated. The amount of bound HRP conjugate was reversely proportional to hormone concentration in the samples. After addition of the substrate solution, the intensity of color developed was reversely proportional to the concentration of hormone in the sample. The plate was read at 450 nm using a BioTek Instruments EL800 Microplate Reader (BioTek Instruments, Inc., Winooski, VT, USA).

### Assessment of sperm concentration, motility and abnormality

Sperm suspension was obtained by mincing the left testes in a prewarmed saline (37 °C). The percentage of total and progressive motility was estimated microscopically according to the method of Morrissey et al. (1988). A total of at least 200 sperm cells were evaluated from four different fields in each sample. The sperm count was determined using a hemocytometer (Freud & Carol, 1964). Sperm abnormality was recorded by using one or two drops of previously warmed (37 °C) eosin-nigrosin stain (Evans & Maxwell, 1987).

### Data analysis

Statistical calculations were performed using SPSS for Windows (version 17; SPSS Inc., Chicago, IL, USA). The results were expressed as the mean ± S.E. (standard error) and statistical significance (*P* ≤ 0.05) was evaluated by one-way analysis of variance (ANOVA) followed by the least significant difference (LSD) post hoc test.

## Results

### Effects of rutin on CCl_4_-induced hepatotoxicity

As shown in Table 1, CCl_4_ administration induced significant increase in serum ALP (157.6%), AST (191.8%) and ALT (160.4%) as compared to control values. Rutin treatment with CCl_4_ significantly reduced ALP by −28.7%, AST by −55.3% and ALT by -39.1% when compared to CCl_4_ group. In addition, total bilirubin significantly increased (100%) while protein and albumin levels significantly decreased (−37.8% and −55.9%, respectively) in CCl_4_ group as compared to control. However, rats in the Rutin + CCl_4_ group had significantly lower levels of total bilirubin (−25%), and higher levels of total protein (41.3%) and albumin (80%) than those in the CCl_4_ group.

**Table 1:** Effect of rutin on serum liver markers in male rats intoxicated with CCl_4_.

	**ALT (U/L)**	**AST (U/L)**	**ALP (U/L)**	**Bilirubin (gm/dl)**	**Protein (gm/dl)**	**Albumin (gm/dl)**
Control	32.8^a^± 1.50	56.4^a^± 1.50	36.8^a^± 2.40	0.4^a^± 0.01	7.4^a^± 0.30	3.4^a^± 0.30
CCl_4_	85.4^b^± 4.30	164.6^b^± 3.40	94.8^b^± 2.60	0.8^b^± 0.03	4.6^b^± 0.20	1.5^b^± 0.30
Rutin	30.2^a^± 2.30	56.0^a^± 2.90	38.4^a^± 2.70	0.5^a^± 0.01	7.6^a^± 0.20	3.5^a^± 0.20
Rutin + CCl_4_	52.0^c^± 2.90	73.6^c^± 1.10	67.6^c^± 3.00	0.6^c^± 0.02	6.5^c^± 0.30	2.7^c^± 0.20

Data are expressed as mean ± SE of *n* = 5 rats/group. Different superscripts within the same column indicate significant differences between the groups (*p* ≤ 0.05). Treatments: control (vehicles); CCl_4_ (3 ml/kg b.w., by intraperitoneal route); rutin (70 mg/kg b.w., by gavage); both rutin + CCl_4_ co-treated group received CCl_4_ and rutin at the same doses twice a week for 4 weeks.

AST aspirate transaminase ALT alanine transaminase ALP alkaline phosphatase

### Effects of rutin on CCl_4_-induced changes in the levels of serum renal function test parameters

**Figure 1:**
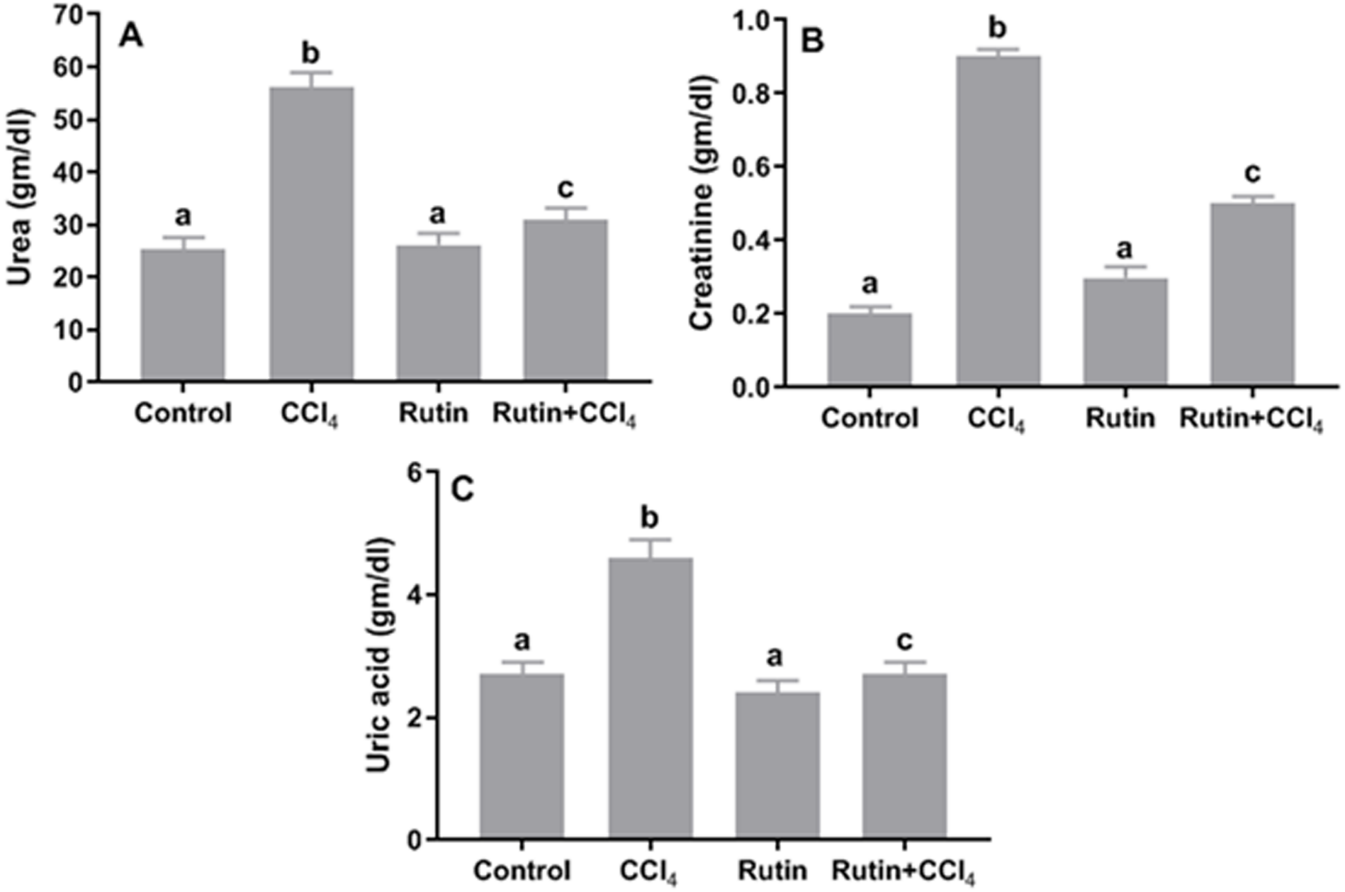
Serum kidney markers in male rats after administration of CCl_4_ and/or rutin. (A) Urea, (B) creatinine and (C) uric acid. Data are expressed as the mean ± SE of *n* = 5 rats/group. Bars with different letters show significant differences between the groups (*p* ≤ 0.05). Treatments: Control (vehicles); CCl_4_ (3 ml/kg b.w., by intraperitoneal route); rutin (70 mg/kg b.w., by gavage); both Rutin + CCl_4_ co-treated group received CCl_4_ and rutin at the same doses twice a week for 4 weeks.

The levels of urea, creatinine and uric acid significantly increased in CCl_4_ group (122.2%, 350%, 70.4%, respectively) as compared with the control group values (Fig. 1). Conversely, Rutin + CCl_4_ group manifested significant declines in urea (−45%), creatinine (−44.4%) and uric acid (−41.3%) as compared to the CCl_4_ group.

### Effects of rutin on CCl_4_-induced liver histopathology

**Figure 2:**
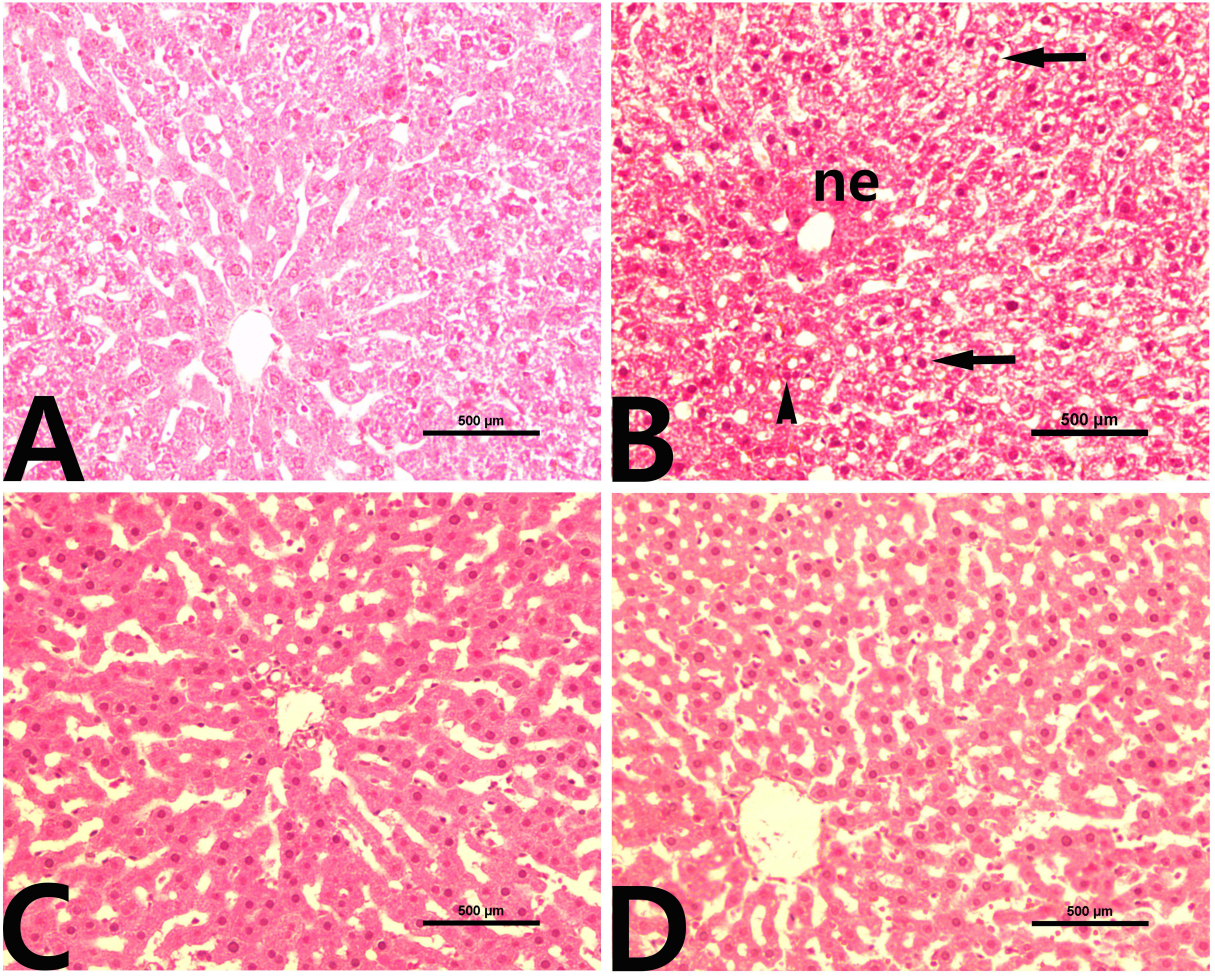
Liver histopathology in male rats after administration of CCl_4_ and/or rutin. (A) Control; (B) CCl_4_; (C) rutin; and (D) Rutin + CCl_4_. Note centrilobular necrosis (ne), fatty degeneration (arrows) and inflammation (arrowhead) in the liver parenchyma. Treatments: Control (vehicles); CCl_4_ (3 ml/kg b.w., by intraperitoneal route); rutin (70 mg/kg b.w., by gavage); both rutin + CCl_4_ co-treated group received CCl_4_ and rutin at the same doses twice a week for 4 weeks.

Liver tissue from control rats (Fig. 2A) presented a normal histological image and hepatocyte structure. In the group treated with CCl_4_, we noted massive centrilobular necrosis with hepatocyte degeneration, vacuolar fatty change and mild mononuclear cell infiltration (Fig. 2B). In the rutin group, the histological appearances of liver lobules and hepatocytes were normal (Fig. 2C). The livers of rats cotreated with rutin showed minimal hepatocellular necrosis and maintained lobular architecture and hepatocyte structure with no evidence of inflammatory cell infiltration or fatty change (Fig. 2D).

### Effects of rutin on CCl_4_-induced renal histopathology

**Figure 3:**
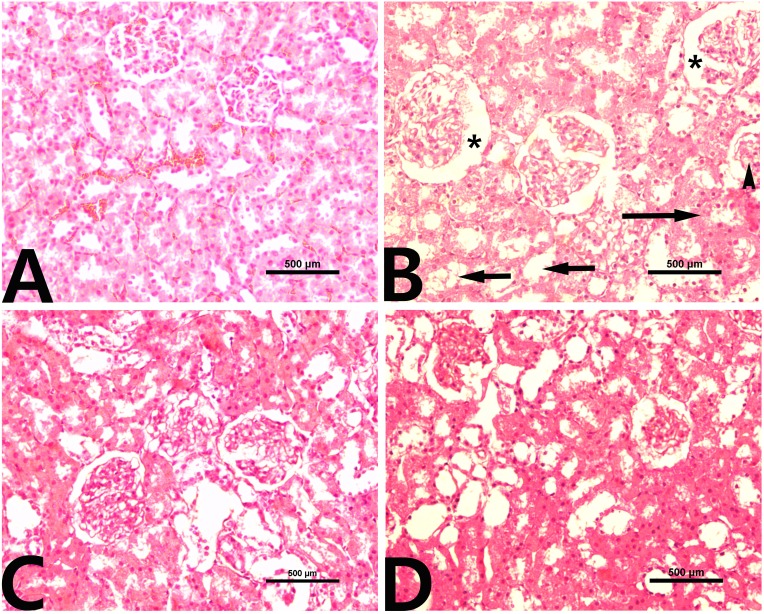
Renal histopathology in male rats after administration of CCl_4_ and/or rutin. (A) Control; (B) CCl_4_; (C) rutin; and (D) Rutin + CCl_4_. Note dilatation of Bowman’s space (*), glomerular atrophy (arrowhead), tubular dilatation (short arrows) and inflammatory cell infiltration (long arrow). Treatments: Control (vehicles); CCl_4_ (3 ml/kg b.w., by intraperitoneal route); rutin (70 mg/kg b.w., by gavage); both Rutin + CCl_4_ co-treated group received CCl_4_ and rutin at the same doses twice a week for 4 weeks.

Normal histological architecture of the kidney was observed in the control group (Fig. 3A). CCl_4_ administration caused distinct morphologic changes, especially in the renal cortex (Fig. 3B). Glomerular changes were focal and included mild dilation of Bowman’s space with an adhesion between the visceral and parietal layers of Bowman’s capsule and glomerular atrophy. In addition, renal tubules were severely dilated, and their epithelial cells tended to be flattened. Moreover, inflammatory cell infiltration was also detected in the renal interstitium. The kidneys of rats treated with rutin alone showed no histological evidence of nephrotoxicity (Fig. 3C). The group of rats cotreated with rutin reduced the CCl_4_-induced renal lesions (Fig. 3D).

### Effects of rutin on CCl_4_-induced changes in oxidant-antioxidant system parameters

**Figure 4:**
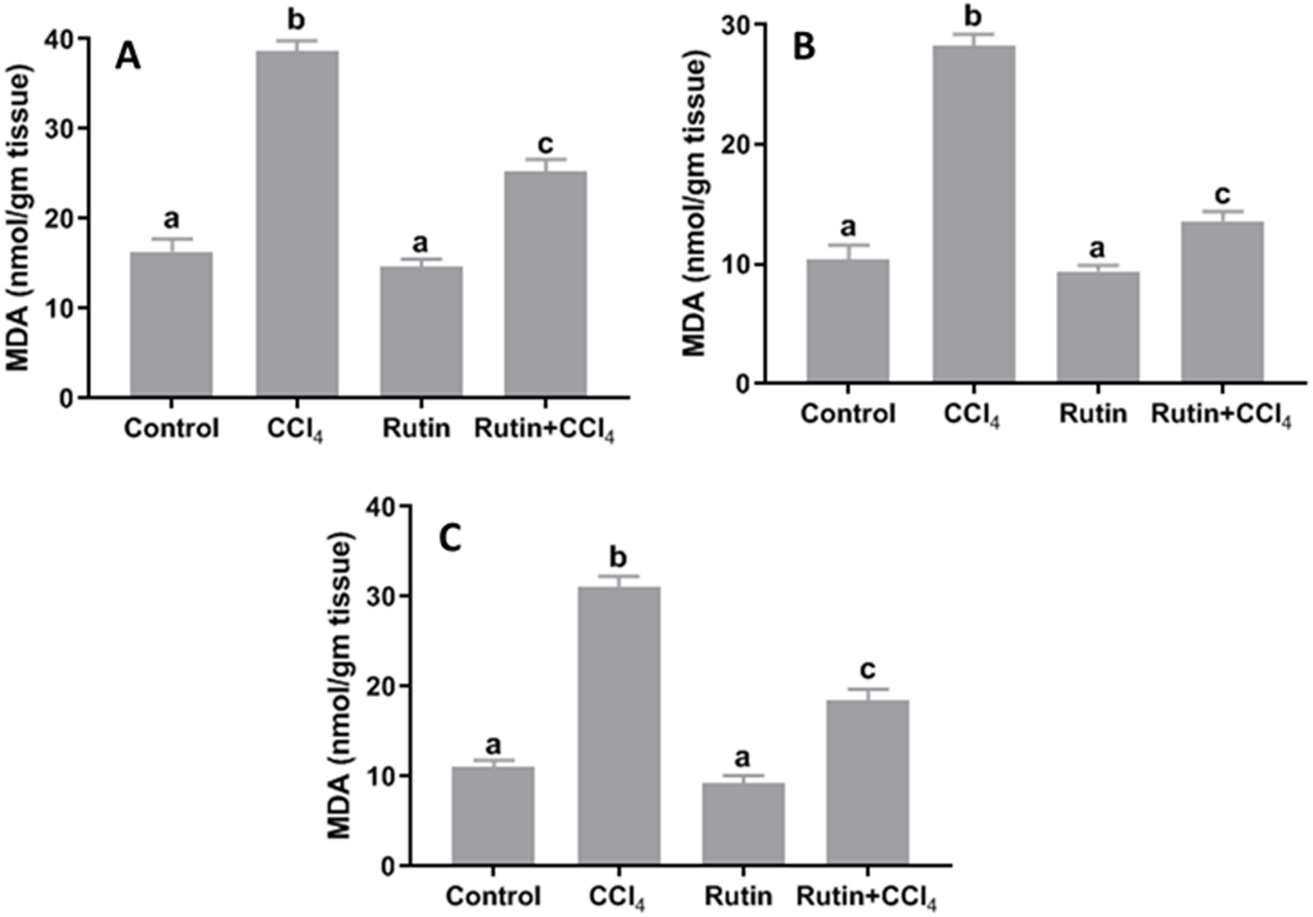
Tissue malondialdehyde (MDA) levels in male rats after administration of CCl_4_ and/or rutin. (A) Liver; (B) Kidney; and (C) Testis. Data are expressed as the mean ± SE of *n* = 5 rats/group. Bars with different letters show significant differences between the groups (*p* ≤ 0.05). Treatments: Control (vehicles); CCl_4_ (3 ml/kg b.w., by intraperitoneal route); rutin (70 mg/kg b.w., by gavage); both rutin + CCl_4_ co-treated group received CCl_4_ and rutin at the same doses twice a week for 4 weeks.

**Figure 5:**
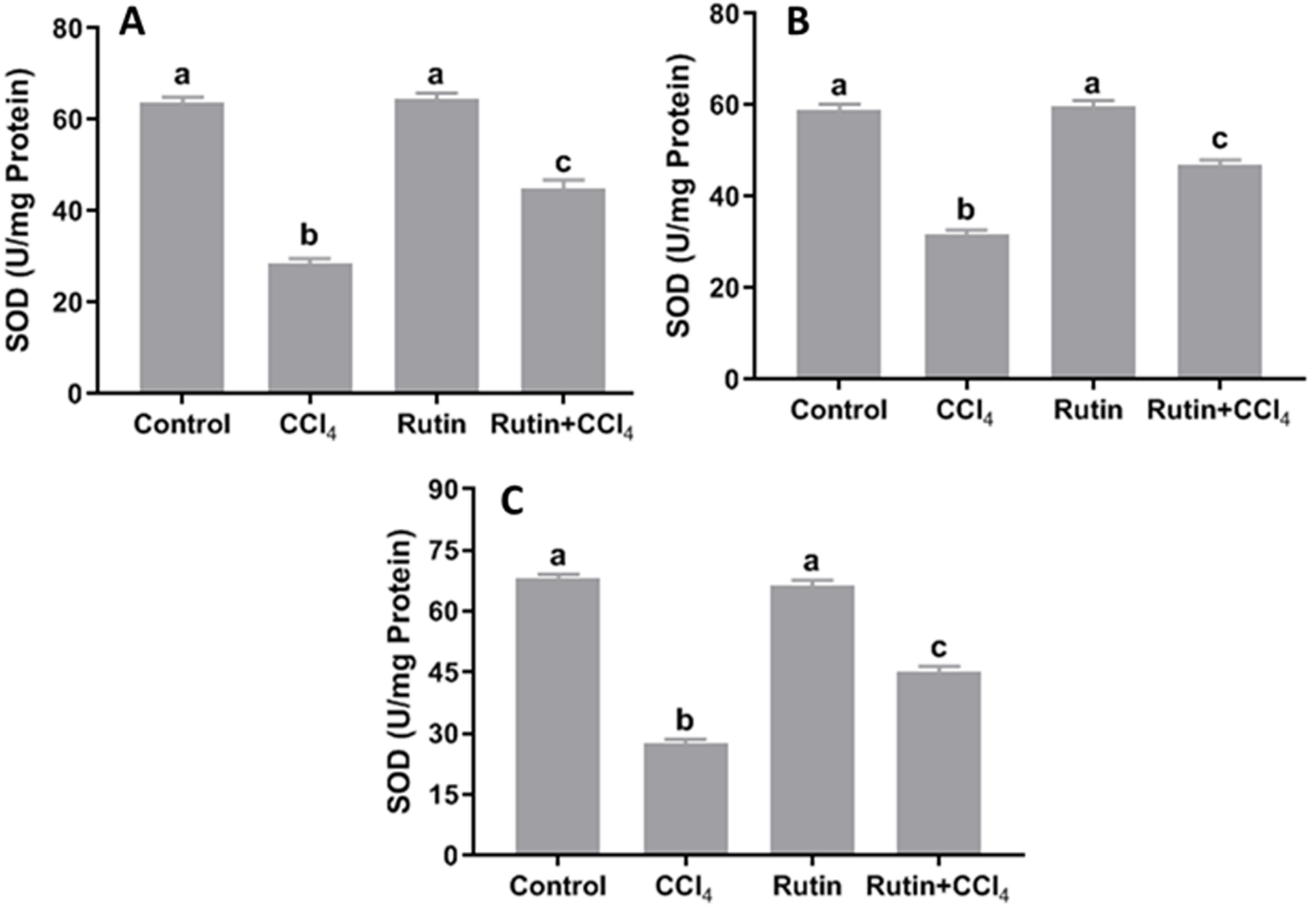
Tissue superoxide dismutase (SOD) levels in male rats after administration of CCl_4_ and/or rutin. (A) Liver; (B) Kidney; and (C) Testis. Data are expressed as the mean ± SE of *n* = 5 rats/group. Bars with different letters show significant differences between the groups (*p* ≤ 0.05). Treatments: Control (vehicles); CCl_4_ (3 ml/kg b.w., by intraperitoneal route); rutin (70 mg/kg b.w., by gavage); both rutin + CCl_4_ co-treated group received CCl_4_ and rutin at the same doses twice a week for 4 weeks.

**Figure 6:**
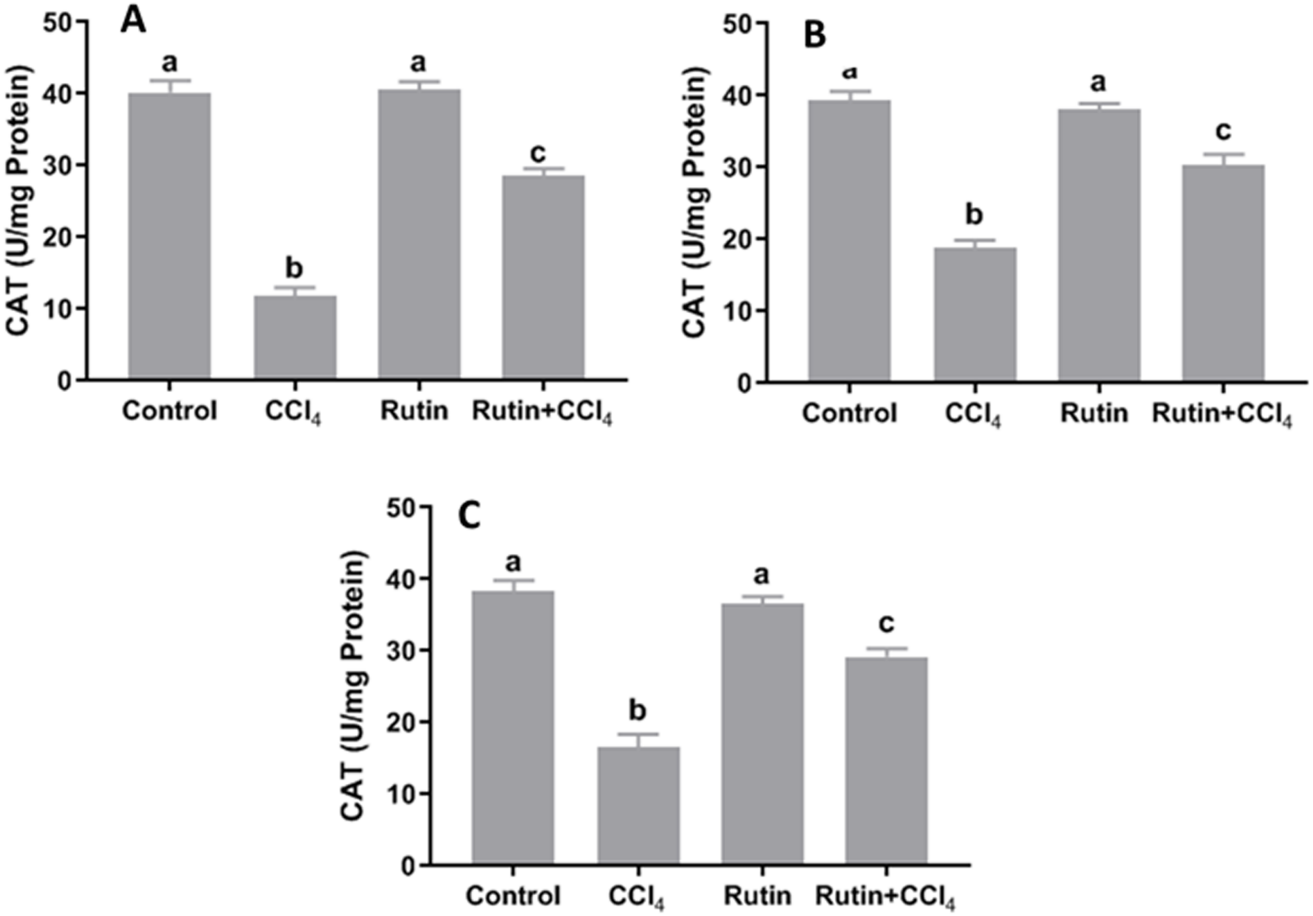
Tissue catalase (CAT) level in male rats after administration of CCl_4_ and/or rutin. (A) Liver; (B) Kidney; and (C) Testis. Data are expressed as the mean ± SE of *n* = 5 rats/group. Bars with different letters show significant differences between the groups (*p* ≤ 0.05). Treatments: Control (vehicles); CCl_4_ (3 ml/kg b.w., by intraperitoneal route); rutin (70 mg/kg b.w., by gavage); both rutin + CCl_4_ co-treated group received CCl_4_ and rutin at the same doses twice a week for 4 weeks.

**Figure 7:**
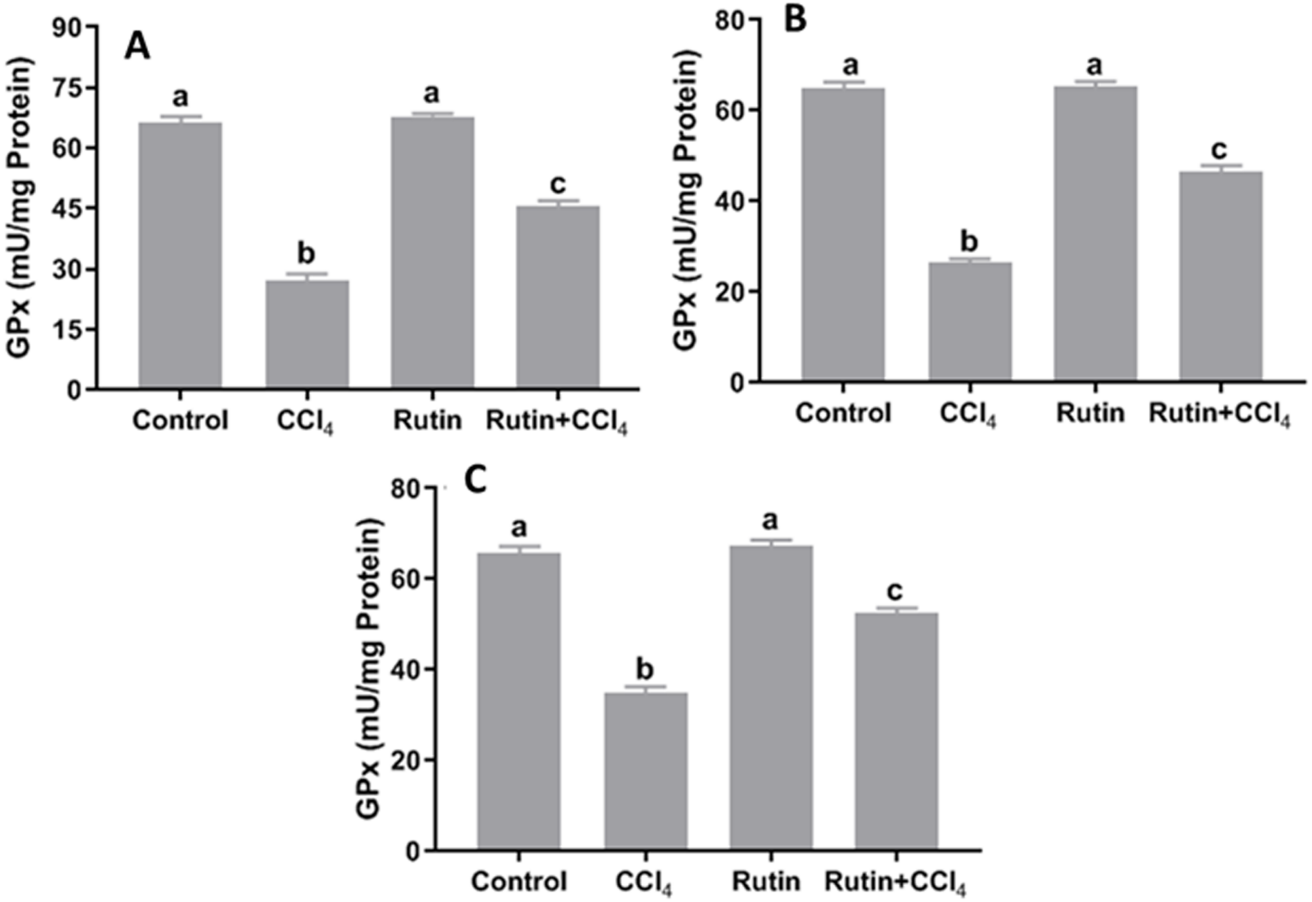
Tissue glutathione peroxidase (GPx) levels in male rats after administration of CCl_4_ and/or rutin. (A) Liver; (B) Kidney; and (C) Testis. Data are expressed as the mean ± SE of *n* = 5 rats/group. Bars with different letters show significant differences between the groups (*p* ≤ 0.05). Treatments: Control (vehicles); CCl_4_ (3 ml/kg b.w., by intraperitoneal route); rutin (70 mg/kg b.w., by gavage); both Rutin + CCl_4_ co-treated group received CCl_4_ and rutin at the same doses twice a week for 4 weeks.

Data presented for liver, kidney and testis showed that CCl_4_ administration significantly increased the lipid peroxidation marker, MDA (135.4%, 171.2% and 181.8%, respectively) (Fig. 4) and significantly decreased the activities of SOD (−55.5%, −45.91% and −59.41%, respectively) (Fig. 5), CAT (−70.6%, −52.3% and −56.8%, respectively) (Fig. 6) and GPx (−59%, −59.1% and −47.1%, respectively) (Fig. 7) when compared to control corresponding values. The combination of rutin with CCl_4_ significantly inhibited MDA levels in liver (−34.7%), kidney (−51.8%), and testis (−40.7%) compared with those of CCl_4_-treated rats. In contrast, co-treatment by rutin resulted in significant enhancement of hepatic, renal and testicular enzymatic activities, i.e., SOD (58.5%, 47.1%, and 63%, respectively), CAT (142.4%, 61.7%, 75.9%, respectively) and GPx (67.7%, 75.2%, and 50.6%, respectively) *versus* the CCl_4_-treated group values.

### Effects of rutin on CCl_4_-induced changes in serum lipid content

**Figure 8:**
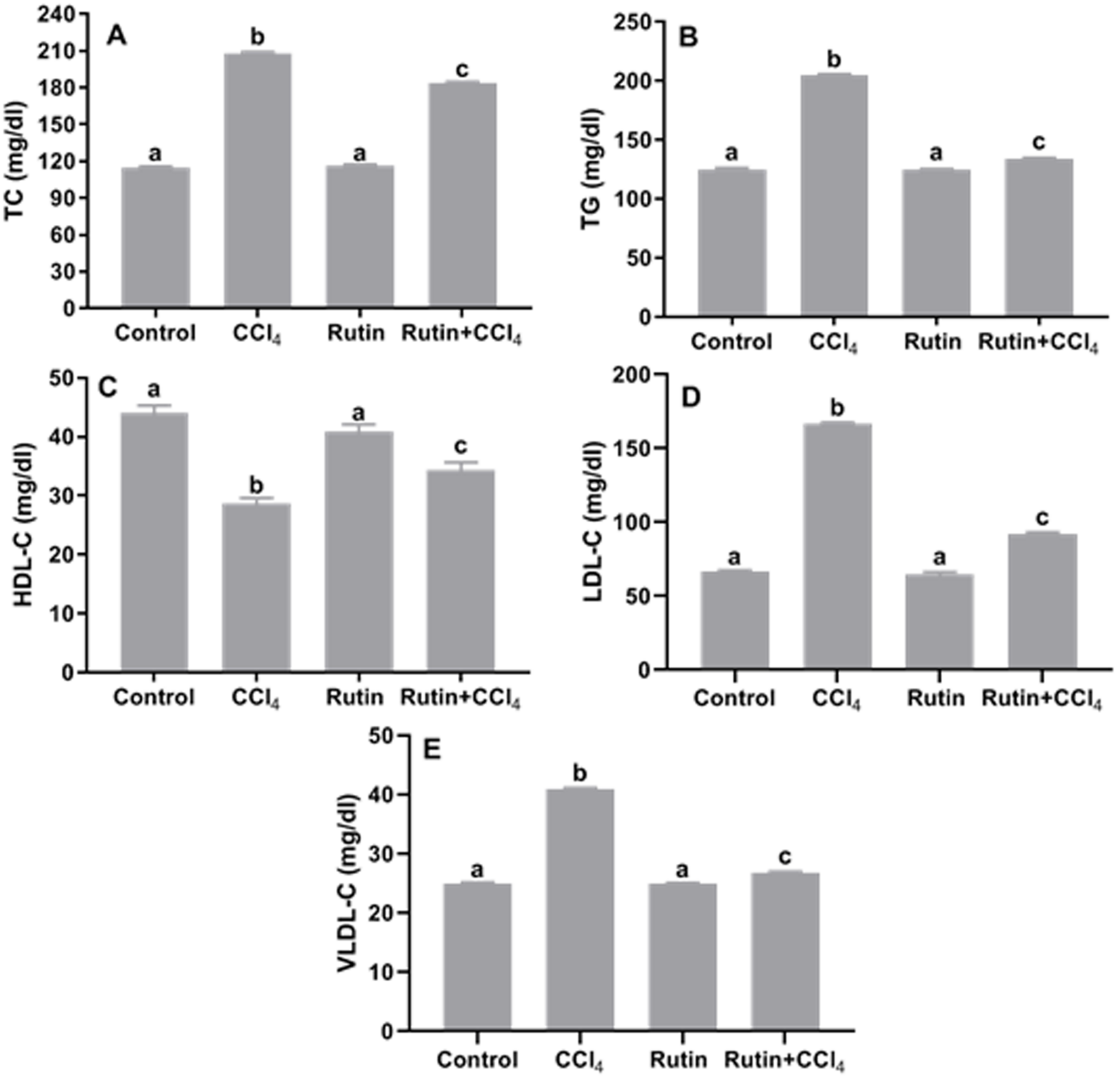
Serum lipid profile in male rats after administration of CCl_4_ and/or rutin. (A) TC: total cholesterol, (B) TG: triglycerides, (C) HDL-C: high-density lipoprotein-cholesterol, (D) low-density lipoprotein-cholesterol and (E) VLDL-C: very-low-density lipoprotein-cholesterol. Data are expressed as the mean ± SE of *n* = 5 rats/group. Bars with different letters show significant differences between the groups (*p* ≤ 0.05). Treatments: Control (vehicles); CCl_4_ (3 ml/kg b.w., by intraperitoneal route); rutin (70 mg/kg b.w., by gavage); both rutin + CCl_4_ co-treated group received CCl_4_ and rutin at the same doses twice a week for 4 weeks.

The lipid profile presented in Fig. 8 showed significant increase in the levels of TC (81.2%), TG (63.4%), LDL-C (150.5%) and VLDL-C (63.6%) and significant decrease in HDL-C (−35%) in the CCl_4_ group as compared to the corresponding control values. However, treatment with rutin remarkably decreased TC (−11.5%), TG (−34.5%), LDL-C (−44.7%) and VLDL-C (−34.5%) levels and increased HDL-C (20.3%) as compared to CCl_4_ treated rats.

### Effects of rutin on CCl_4_-induced changes in serum testosterone, LH and FSH

**Figure 9:**
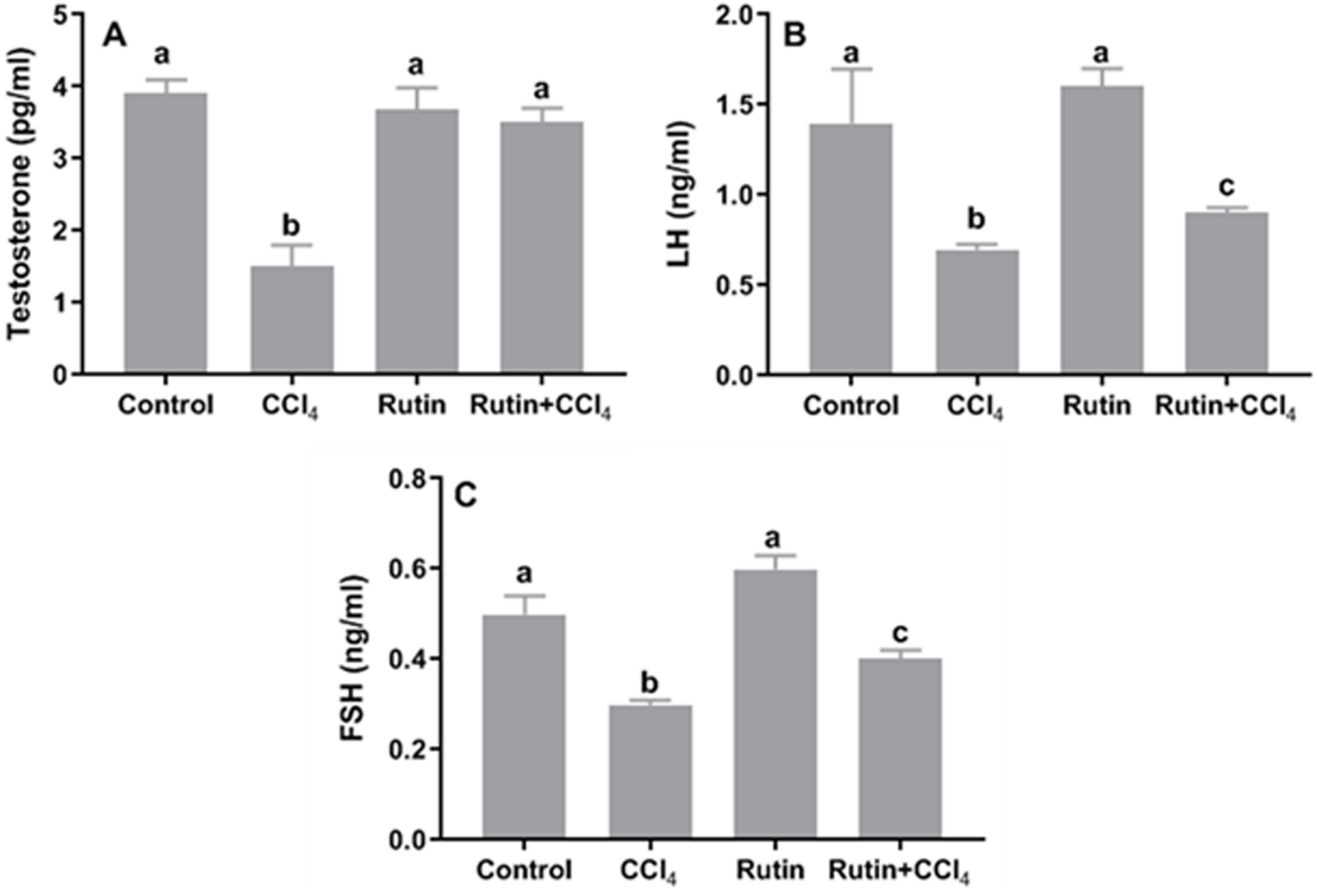
Serum reproductive hormones in male rats after administration of CCl_4_ and/or rutin. (A) Testosterone, (B) LH and (C) FSH. Data are expressed as the mean ± SE of *n* = 5 rats/group. Bars with different letters show significant differences between the groups (*p* ≤ 0.05). Treatments: Control (vehicles); CCl_4_ (3 ml/kg b.w., by intraperitoneal route); rutin (70 mg/kg b.w., by gavage); both rutin + CCl_4_ co-treated group received CCl_4_ and rutin at the same doses twice a week for 4 weeks.

Treatment of rats with CCl_4_ led to a significant decrease in serum levels of testosterone (−61.5%), LH (−50%) and FSH (−40%) when compared to the corresponding control levels. While, these hormones were restored in Rutin + CCl_4_ group through significant increasing of testosterone (133.3%; normalization), LH (28.6%) and FSH (33.3%) relative to their corresponding values in CCl_4_ treated group (Fig. 9).

**Figure 10:**
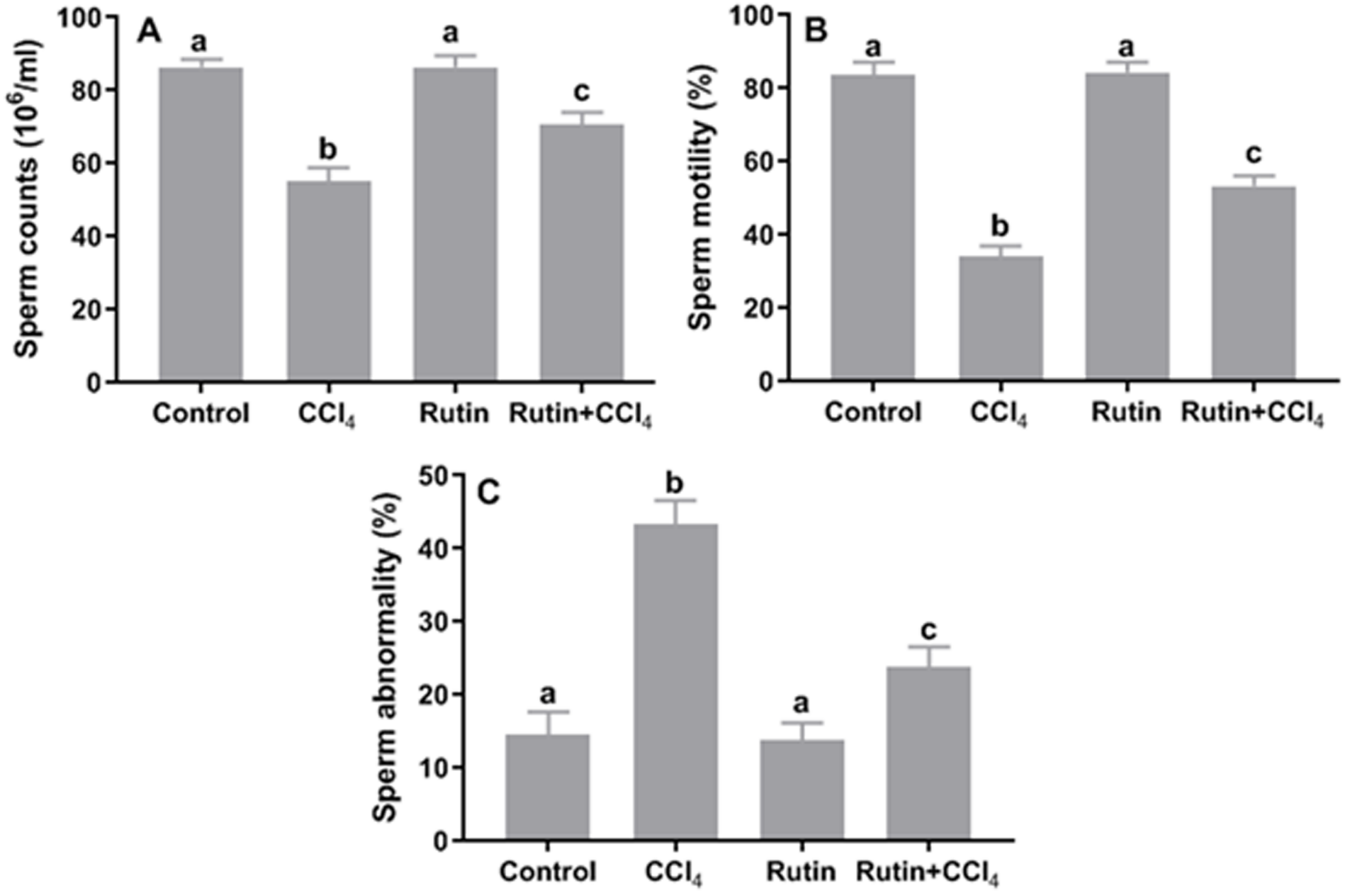
Sperm parameters in male rats after administration of CCl_4_ and/or rutin. (A) Sperm counts, (B) sperm motility and (C) sperm abnormality. Data are expressed as the mean ± SE of *n* = 5 rats/group. Bars with different letters show significant differences between the groups (*p* ≤ 0.05). Treatments: Control (vehicles); CCl_4_ (3 ml/kg b.w., by intraperitoneal route); rutin (70 mg/kg b.w., by gavage); both rutin + CCl_4_ co-treated group received CCl_4_ and rutin at the same doses twice a week for 4 weeks.

### Effects of rutin on CCl_4_-induced changes in sperm parameters

Relative to control, CCl_4_ administration caused significant decrease in sperm count (−36.3%) and sperm motility (−59.2%) and significant increase in the percentage of sperm abnormality (197.3%) (Fig. 10). However, simultaneous supplementation of CCl_4_ plus rutin significantly increased sperm count (28.4%) and sperm motility (56.1%), and decreased sperm abnormality (−45.2%) compared to rats treated with CCl_4_ alone.

## Discussion

CCl_4_ is known for its hepatotoxicity and nephrotoxicity. The degree of toxicity depends on the overproduction of reactive oxygen species (ROS), oxidative injury and the inflammatory process. CCl_4_ intoxication generates free radicals such as nitric oxide and peroxynitrite that trigger a cascade of events resulting in liver and kidney damage in rats (Khan & Zehra, 2013; Jan & Khan, 2016). Accordingly, we found significant elevation of serum ALP, AST, ALT, and bilirubin levels, while protein and albumin levels were significantly decreased in the CCl_4_-treated group. These results are indicators of hepatocyte dysfunction, cellular leakage and loss of functional integrity of the cell membrane in the liver, as previously reported (Khan, Khan & Sahreen, 2012). Rutin administration remarkably prevented the hepatic injury caused by CCl_4_. Rutin maintains liver enzyme homeostasis by acting as a membrane-stabilizing agent that inhibits leakage of enzymes due to its polyphenolic natural effects (Khan, Khan & Sahreen, 2012; Gelen et al., 2017). In addition, rutin enhances antioxidant gene expression and decreases hepatic DNA damage in the oxidative stress pathway initiated by CCl_4_ administration (Hafez et al., 2014). Recently, rutin treatment was proven to have hepatoprotective effects against STZ-induced type 1 diabetic conditions in mice (Li et al., 2018). Reddy et al. (2017) showed the superiority of rutin over silymarin in restoring the pathological alterations in paracetamol-induced hepatotoxicity in Wistar albino rats.

Administration of CCl_4_ causes nephrotoxicity as indicated by elevation in serum levels of urea, creatinine and uric acid. The high levels of creatinine and urea are indicators of severe damage to the structural integrity of nephrons. Coadministration of rutin significantly reduced these diagnostic markers. Several researchers have reported that rutin can modulate nephrotoxicity through its regulatory effect on apoptotic pathways including inhibition of several activation of caspases (Alhoshani et al., 2017; Khajevand-Khazaei et al., 2018). Radwan & Abdel Fattah (2017) indicated that the nephroprotective effect of rutin might be valuable in improving the therapeutic index of cisplatin.

Oxidative stress is a term that refers to altered cellular redox balance. Our results revealed a significant increase in the LPO marker (i.e., MDA) and a significant decrease in SOD, CAT and GPx enzyme activities in rat tissues following CCl_4_ application. This is in agreement with previous studies (Szymonik-Lesiuk et al., 2003; Alqasoumi & Abdel-Kader, 2012; Jan & Khan, 2016; Noureen et al., 2017). Treatment with rutin significantly decreased MDA and increased SOD, CAT and GPx activities. Rutin acts as a master redox switch through Nrf2 activation and iNOS suppression (Singh et al., 2018). The antioxidant activities of rutin have been found to be due to (1) its chemical structure, which can directly scavenge ROS (Hanasaki, Ogawa & Fukui, 1994); (2) its ability to increase the production of GSH and the cellular defense system (Shenbagam & Nalini, 2011); and (3) its inhibitory effect on xanthine oxidase, which is involved in generating ROS (Kostić et al., 2015).

The current results implied that CCl_4_ affected testicular spermatogenic function. We found a significant decrease in serum levels of testosterone, LH, and FSH compared with control values. LPO induced by CCl_4_ may affect the testis response to FSH and LH and subsequently reduce testosterone secretion (Rafiee et al., 2016). The oxidative stress of the testicular germinal layer is a central cause of the intrinsic apoptotic cascade, which can trigger mitochondrial ROS generation and provoke hypogonadism (Aitken et al., 2014). In addition, CCl_4_ reduced the secretion of testosterone, FSH and LH in rats through direct effects on the central nervous system and Leydig cells and/or indirectly by affecting the hypothalamic-pituitary estrogen receptor axis (Okolo, Siminialayi & Orisakwe, 2016; Noureen et al., 2017). CCl_4_ enhanced cellular degeneration by increasing the production of H_2_O_2_ and nitrite, which resulted in infertility/testicular dysfunction (Sahreen, Khan & Khan, 2013; Ojo et al., 2016). In turn, H_2_O_2_ might diffuse across the membrane and affect the sperm vital enzymes, thereby resulting in decreased sperm motility (Gautam et al., 2006; Khan & Ahmed, 2009; Sun et al., 2017). According to previous studies, antioxidants can help prevent and repair cell damage, so they may have an impact on the semen quality parameters and fertility potential (e.g., Dang et al., 2017). In this context, Aksu et al. (2017) showed that the antioxidant potential of rutin mitigated sperm damage, testicular degeneration and apoptosis induced by cisplatin treatment in adult male rats. Further research with rutin has shown the capacity to prevent cadmium-induced reproductive toxicity in rats, maintaining both antioxidants and testicular androgenic enzymes (Abarikwu, Iserhienrhien & Badejo, 2013).

For the lipid profile, the serum levels of total cholesterol, TG, LDL-C, and VLDL-C showed remarkable increases in CCl_4_-treated rats. In fact, high serum levels of cholesterol are associated with testicular dysfunction (Kalender et al., 2013) and decreased sperm quality (Lassoued et al., 2018). Diets with low cholesterol and high antioxidative agents can help to improve spermatogenesis and consequently promote male fertility (Khorrami et al., 2015). Previous studies have indicated that increased LDL plasma concentration is an independent risk factor for spermatogenesis (Chen et al., 2011). The oxidative conversion of pure LDL to oxidized LDL by free radicals is considered to be involved in cell injury and DNA damage, especially in tissues with a high rate of cell division, such as testis, epididymis and seminal vesicles (Kosola et al., 2013). Furthermore, experimental studies have found that longstanding hyperlipidemia leads to increased lipid oxidation and progression of liver and kidney pathologies (Gyebi, Soltani & Reisin, 2012; Gou et al., 2016). Herein, we found that rutin treatment alleviated the severity of hyperlipidemia in CCl_4_-treated rats. Rutin has hypolipidemic action due to its ability to inhibit lipogenesis and activate the metabolism of fatty acids (Liu et al., 2017). It was suggested that supplementation of quercetin, one of the metabolites of rutin, promotes an increase in faecal sterols, which in turn leads to a decreased absorption of dietary cholesterol as well as lowered plasma and hepatic cholesterol (Bok et al., 2002).

## Conclusions

In the present work, it appears that the administration of rutin contributes to its protective effect against CCl_4_-induced hepatorenal injuries and testicular/fertility disturbances not only through recovery of ROS-mediated oxidative stress but also through marked hypolipidemic activity. Therefore, in the future, oral rutin intake as an adjunct natural therapy may be advisable for male subjects to protect against liver/kidney failure-mediated inhibitory effects on their reproductive function.

##  Supplemental Information

10.7717/peerj.7011/supp-1 Data S1 Raw data applied for statistical analyses and preparation for Table 1, Fig. 1, and Figs. 4–10

## Additional Information and Declarations

### Data Availability

The following information was supplied regarding data availability: The raw measurements are available as a Supplemental File and were used for statistical analysis.
